# Comparison of hesitancy between COVID-19 and seasonal influenza vaccinations within the general Hungarian population: a cross-sectional study

**DOI:** 10.1186/s12889-021-12386-0

**Published:** 2021-12-23

**Authors:** Viktor Dombrádi, Tamás Joó, Gergely Palla, Péter Pollner, Éva Belicza

**Affiliations:** 1 Health Services Management Training Centre, Faculty of Health and Public Administration, Semmelweis University, Budapest, Hungary; 2 Health Security and Cyber Defense Knowledge Centre, Health Services Management Training Centre, Faculty of Health and Public Administration, Semmelweis University, Budapest, Hungary; 3 Hungarian Healthcare Management Association, Budapest, Hungary; 4 MTA-ELTE Statistical and Biological Physics Research Group, Eötvös Loránd Research Network (ELKH), Department of Biological Physics, Eötvös Loránd University, Budapest, Hungary

**Keywords:** COVID-19, SARS-CoV-2, Influenza, Vaccination, Hesitancy, Population

## Abstract

**Background:**

The willingness to get COVID-19 or seasonal influenza vaccines has not yet been thoroughly investigated together, thus, this study aims to explore this notion within the general adult population.

**Methods:**

The responses of 840 Hungarian participants were analysed who took part in a nationwide computer-assisted telephone interviewing. During the survey questions concerning various demographic characteristics, perceived financial status, and willingness to get the two types of vaccines were asked. Descriptive statistics, comparative statistics and word co-occurrence network analysis were conducted.

**Results:**

48.2% of participants were willing to get a COVID-19 vaccine, while this ratio for the seasonal influenza was only 25.7%. The difference was significant. Regardless of how the participants were grouped, based on demographic data or perceived financial status, the significant difference always persisted. Being older than 59 years significantly increased the willingness to get both vaccines when compared to the middle-aged groups, but not when compared to the younger ones. Having higher education significantly elevated the acceptance of COVID-19 vaccination in comparison to secondary education. The willingness of getting any type of COVID-19 vaccine correlated with the willingness to get both influenza and COVID-19. Finally, those who were willing to get either vaccine coupled similar words together to describe their thoughts about a COVID-19 vaccination.

**Conclusion:**

The overall results show a clear preference for a COVID-19 vaccine and there are several similarities between the nature of willingness to get either type of vaccine.

**Supplementary Information:**

The online version contains supplementary material available at 10.1186/s12889-021-12386-0.

## Background

Due to the Coronavirus Disease 2019 (COVID-19) pandemic, caused by the Severe Acute Respiratory Syndrome Coronavirus 2 (SARS-CoV-2), millions of people have been infected, resulting in more than 4.2 million deaths as of early-August 2021 and considerable economic damage was inflicted worldwide as well [1–4]. Furthermore, healthcare workers and those who are most vulnerable are disproportionately more severely affected by this pandemic [5–7]. Nevertheless, since December 2020 several new vaccines have been introduced against COVID-19 which have an efficacy between 70 and 95% [8–11]. Therefore, governments all around the world have initiated a mass vaccination programme for their citizens, starting with essential workers and those who have an increased risk of mortality [12, 13].

These new vaccines also prompted a debate on the questions if a COVID-19 vaccination should be mandatory for the citizens and if those getting a vaccine should get some form of monetary compensation [14–16]. However, regardless of which approach would be most suitable in the long term, the governments of Western countries have decided to protect individual freedom and thus making a COVID-19 vaccination free of charge but voluntary for their citizens [17–19]. This, whatsoever, raises the question of how decision-makers can improve the willingness among the population to receive COVID-19 vaccination [20]. Those who are unwilling or unsure to get a vaccination are considered hesitant. Several studies have investigated the nature of COVID-19 vaccination hesitancy [21–40], and there is a wealth of literature investigating the same issue for seasonal influenza vaccination [41, 42]. However, for both type of vaccinations there is noteworthy inconsistency on what factors influence hesitancy; thus, further investigations are justified.

The relationship between COVID-19 and seasonal influenza was previously explored in a study conducted in the United States, in which they found that while 66.7% of the population was willing to get a COVID-19 vaccine, only 53.0% panned to get the next seasonal influenza vaccine [43]. Also, an Italian longitudinal study reported that the median value of COVID-19 vaccine hesitancy was between 1.0-25.0, while the median value of seasonal influenza vaccine hesitancy was between 50.0-82.0; showing that the Italian population also perceives COVID-19 vaccine much more favourably [44]. Besides knowing that having the last seasonal influenza vaccine predicts COVID-19 vaccine acceptance [39, 45, 46], not much is known regarding the nature of hesitancy considering both vaccine types.

Since vaccine hesitancy could be a major factor of how well the COVID-19 pandemic is tackled globally [47, 48], the aims of this study were to investigate how the intention of the general Hungarian population differs regarding getting a COVID-19 and the next annual seasonal influenza vaccinations and to further investigate what factors influence hesitancy for both type of vaccines.

## Methods

### Design, setting and respondents

The database for our study was provided by the Nézőpont Intézet Ltd. [49], which conducted a nationwide computer-assisted telephone interviewing in Hungary between 5-6th Augusts 2020. Only adult Hungarian citizens could participate. The survey was representative by sex, age (18 and older), education level, type of residence, and NUTS 1 (Nomenclature of Territorial Units for Statistics) region in Hungary. The staff of the Nézőpont Intézet Ltd. originally aimed to reach 1000 participants, and at the end of the survey they managed to get 1013 answers. To ensure that the final sample is representative the company used random-dialling for sampling. All of the Hungarian telephone numbers had the same probability to be the part of this sample. The Nézőpont Intézet Ltd. used quotas (by sex, age, education level, type of residence, and region in Hungary) and weighting (per person not more than 3 and less than 0.5) to ensure that the final sample is representative. The representativeness of the adult Hungarian population was based on the mid-year population estimates provided by the Hungarian Central Statistical Office.

The original aim of the telephone interviewing was to provide data to the Hungarian decision-makers in order to understand the willingness of getting a then possible COVID-19 vaccination and the next seasonal influenza vaccination among the citizens of Hungary.

### Ethical consideration

The study design and protocol were reviewed and approved by the Hungarian Scientific Research and Ethics Committee of the Medical Research Council (protocol code: IV/2288-1/2021/EKU). At the beginning of each and every telephone interview the operators asked the participants to provide consent.

### Measures

The survey asked the participants about their intentions to get a COVID-19 vaccination, and to get the next annually available seasonal influenza vaccination. Answering the questions related to the demographic data were mandatory; however, the respondents had the option not to answer the questions regarding their financial status or the willingness to get any of the two vaccinations. As part of the survey, the respondents had the chance to describe their thoughts about a COVID-19 vaccination by listing 5 free keywords that they considered to be the most relevant related to this issue. At this question, however, most of the participants answered with a mixture of keywords and complete or half sentences. This ‘raw input’ was recorded and later typed into text. Employees of the Nézőpont Intézet Ltd. read all the texts and sorted the answers manually into 26 categories, reflecting the main attitude, or the strongest feeling that can be inferred from the given sentence, half-sentence or keyword. The categories with the corresponding words (translated from Hungarian to English) can be seen in Additional file 1. Furthermore, the ten most commonly used categorical keywords were also classified by the authors of this paper based on their sentiment such as ‘positive’ and ‘negative’.

### Data analyses

As part of the data processing, of the original 1013 participants, only those 840 were included in the statistical and word co-occurrence network analyses who answered both questions regarding a COVID-19 and seasonal influenza vaccines, since the goal of the study was the comparison of these two categories.

For the descriptive statistics frequency and percentage were calculated. McNemar’s test was applied when comparing the willingness of getting a COVID-19 vaccine to that of getting a seasonal influenza vaccine. This was done first for all 840 participants of the study, and later was stratified by the various demographic data and perceived financial status. Univariate and multivariate logistic regression was used to investigate how the various demographic data, perceived financial status, and the willingness to get the other type of vaccination influence the willingness to get COVID-19 and seasonal influenza vaccinations. When calculating the multivariate logistic regression analyses, all demographic characteristics, the perceived financial status, and the willingness to get the other type of vaccine were included as confounders. Furthermore, those who did not answer the question regarding the perceived financial status were excluded from both the McNemar’s tests and the logistic regression analyses. Chi-squared test was utilized when comparing the willingness to get COVID-19 and seasonal influenza vaccinations based on the most common keywords used by the responders when describing their thoughts on a COVID-19 vaccination.

A *P*-value less than 0.05 was considered significant. All the statistical analyses were performed using the SPSS 27 software program (IBM Corp. Released 2020. IBM SPSS Statistics for Windows, Version 27.0).

Finally, the words appearing in the raw free text given by the participants were made subject to a co-occurrence network analysis in order to gain a simple visual representation of the plausible mindset of the respondents [50, 51]. During this process, the stop words (such as ‘a’, ‘and’, etc.) were removed and the remaining words were stemmed. Note that the responses were collected in the Hungarian language, but we show the co-occurrence network with translated phrases. Hence there are words in the network that are handled usually as stop words in English. In addition, the function words (such as ‘within’ or ‘to be’) were not removed as these provide important context that can be used in the relational analysis, and because within the Hungarian language the usage of such words creates a slight difference in meaning. For example, using a ‘be’ in a Hungarian sentence emphasises the existence of something. The co-occurrence between the resulting words was recorded separately for the four groups of participants arising according to the binary choice between the two diseases (COVID-19 or influenza) and the willingness to be vaccinated or the rejection of vaccination against the given disease. Uncertain participants were left out from this analysis. The co-occurrence frequencies between the words were interpreted as link weights, where a minimum threshold of two co-occurrences was set in order to make the analysis more focused on the relevant connections appearing with higher frequencies. The co-occurrence network analysis was conducted with the Cytoscape (U.S. National Institute of General Medical Sciences. Released 2020. Cytoscape, Version 3.8.2) and the NetworkX (NetworkX developers. Released 2020. NetworkX, Version 2.5) software.

## Results

### Sample characteristics

Of the original 1013 participants, 840 (83.0%) answered both questions related to vaccination (Table 1). Of the latter, 449 (53.5%) were female, most of them were 60 years old or older (*n* = 269; 32.0%), and less than a third of them had a higher education (*n* = 255; 30.3%). In addition, the majority of them were from non-county capital cities (*n* = 290; 34.5%) and from the Great Plain and North region of Hungary (*n* = 329; 39.3%). Regarding their perceived financial status, 366 (43.6%) of the participants answered that they need to schedule their expenses, while 10 (1.2%) refused to answer this question.

**Table 1:** Demographic characteristics of the respondents

	All respondents		Answered both vaccination questions	
	n	%	n	%
**Sex**				
Female	550	54.3	449	53.5
Male	463	45.7	391	46.5
**Age**				
18-29	129	12.7	111	13.2
30-39	166	16.4	140	16.7
40-49	207	20.4	182	21.7
50-59	169	16.7	138	16.4
60 or older	342	33.8	269	32.0
**Education**				
Primary	336	33.2	283	33.7
Secondary	360	31.3	302	36.0
Higher	317	35.5	255	30.3
**Residence**				
Budapest (national capital)	193	19.1	151	18.0
City (county capital)	210	20.7	178	21.2
City (other)	352	34.7	290	34.5
Village	258	25.5	221	26.3
**Region (NUTS 1)**				
Central Hungary (HU1)	316	31.2	256	30.5
Transdanubia (HU2)	300	29.6	255	30.4
Great Plain and North (HU3)	397	39.2	329	39.3
**Perceived financial status**				
Have no financial problems whatsoever	155	15.3	135	16.1
Needs to schedule the expenses	456	45.0	366	43.6
The monthly income is just enough	265	26.2	220	26.2
Lives month by month, lacks adequate income	92	9.1	81	9.6
Lives in hardship	32	3.1	28	3.3
No answer	13	1.3	10	1.2
**OVERALL**	1013	100	840	100

*NUTS* Nomenclature of Territorial Units for Statistics, *HU* Hungary

### Responses of the participants

Of the participants nearly half of them rejected both type of vaccination (*n* = 406; 48.3%), around one-fourth was only willing to get a COVID-19 vaccine (*n* = 218; 26.0%), a minority was only willing to get the next seasonal influenza vaccine (*n* = 29; 3.5%), and 22.3% (*n* = 187) were willing to get both vaccines (Table 2). Overall, nearly half of the participants (*n* = 405; 48.2%) stated that they were open to get a COVID-19 vaccine, while only 216 (25.7%) made the same statement regarding seasonal influenza. The difference was significant (*P* < 0.001). Furthermore, regardless of how the participants were grouped based on demographic data or perceived financial status the significant difference always persisted (P < 0.001).

**Table 2:** Willingness among the respondents to get COVID-19 or seasonal influenza vaccination

	Neither vaccination		Only COVID-19 vaccination		Only seasonal influenza vaccination		Both vaccination		COVID-19 vs. seasonal influenza (***P***-value)
	N	%	N	%	N	%	N	%	
**Sex**									
Female	222	49.3	113	25.3	15	3.3	99	22.0	< 0.001*
Male	184	47.1	105	26.9	14	3.6	88	22.5	< 0.001*
**Age**									
18-29	49	44.1	27	24.3	6	5.4	29	26.1	< 0.001*
30-39	74	52.9	42	30.0	7	5.0	17	12.1	< 0.001*
40-49	108	59.3	44	24.2	1	0.5	29	15.9	< 0.001*
50-59	75	54.3	39	28.3	5	3.6	19	13.8	< 0.001*
60 or older	100	37.2	66	24.5	10	3.7	93	34.6	< 0.001*
**Education**									
Primary	123	43.5	73	25.8	10	3.5	77	27.2	< 0.001*
Secondary	169	56.0	67	22.2	12	4.0	54	17.9	< 0.001*
Higher	114	44.7	78	30.6	7	2.7	56	22.0	< 0.001*
**Residence**									
Budapest (national capital)	62	41.4	47	31.1	6	4.0	36	23.8	< 0.001*
City (county capital)	99	55.6	37	20.8	7	3.9	35	19.7	< 0.001*
City (other)	153	52.8	63	21.7	10	3.4	64	22.1	< 0.001*
Village	92	41.6	71	32.1	6	2.7	52	23.5	< 0.001*
**Region (NUTS 1)**									
Central Hungary (HU1)	117	45.7	74	28.9	10	3.9	55	21.5	< 0.001*
Transdanubia (HU2)	135	52.9	61	23.9	8	3.1	51	20.0	< 0.001*
Great Plain and North (HU3)	154	46.8	83	25.2	11	3.3	81	24.6	< 0.001*
**Perceived financial status**									
Have no financial problems whatsoever	62	45.9	45	33.3	5	3.7	23	17.0	< 0.001*
Needs to schedule the expenses	182	49.7	102	27.9	9	2.5	73	19.9	< 0.001*
The monthly income is just enough	102	46.4	49	22.3	8	3.6	61	27.7	< 0.001*
Lives month by month. Lacks adequate income	43	53.1	13	16.0	4	4.9	21	25.9	< 0.001*
Lives in hardship	13	46.4	6	21.4	1	3.6	8	28.6	< 0.001*
**OVERALL**	406	48.3	218	26.0	29	3.5	187	22.3	< 0.001*

*NUTS* Nomenclature of Territorial Units for Statistics, *HU* Hungary, *COVID-19* Coronavirus Disease 2019, *Significant findings (*P* < 0.05)

### Comparative statistical analysis of COVID-19 vaccination

The results of both the univariate and multivariate logistic regression (Table 3) revealed that the 40-49 (*P* = 0.039; AOR = 0.626; 95%CI = 0.401-0976) and the 50-59 age groups (*P* = 0.048; AOR = 0.613; 95%CI = 0.377-0.995) were significantly less willing to get COVID-19 vaccination compared to those being 60 years old or older. Having only a secondary education also significantly lowered the willingness (*P* = 0.011; AOR = 0.597; 95%CI = 0.401-0.888) compared to those having a higher education. The residence of the participants was also a major influencing factor. Those living in a city (county capital: *P* = 0.001; AOR = 0.454; 95%CI = 0.282-0.732; other: *P* = 0.002; AOR = 0.519; 95%CI = 0.344-0.783) were less willing to get a COVID-19 vaccine, than those living in a village. However, this difference was not significant when in the capital city Budapest was compared to villages (*P* = 0.562; AOR = 0.831; 95%CI = 0.445-1.553). Sex, region and perceived financial status did not significantly influence the willingness to get this vaccine. Finally, those who are willing to get a seasonal influenza vaccine are significantly more open to getting COVID-19 vaccination as well (*P* < 0.001; AOR = 12.857; 95%CI = 8.161-20.254).

**Table 3:** Logistic regression analyses of the differences in the willingness to get COVID-19 vaccination

Factors	Univariate analysis				Multivariate analysis			
	OR	***P***-value	95% CI		AOR	***P***-value	95% CI	
**Sex**								
Female	0.918	0.535	0.700	1.204	0.884	0.451	0.642	1.218
Male	1	–	–	–	1	–	–	–
**Age**								
18-29	0.704	0.122	0.452	1.099	0.653	0.122	0.380	1.121
30-39	0.504	0.001*	0.333	0.763	0.656	0.089	0.403	1.067
40-49	0.463	< 0.001*	0.316	0.680	0.626	0.039*	0.401	0.976
50-59	0.502	0.001*	0.331	0.761	0.613	0.048*	0.377	0.995
60 or older	1	–	–	–	1	–	–	–
**Education**								
Primary	1.018	0.916	0.726	1.429	0.902	0.646	0.581	1.401
Secondary	0.604	0.003*	0.431	0.845	0.597	0.011*	0.401	0.888
Higher	1	–	–	–	1	–	–	–
**Residence**								
Budapest (national capital)	0.973	0.896	0.641	1.474	0.831	0.562	0.445	1.553
City (county capital)	0.541	0.003*	0.363	0.807	0.454	0.001*	0.282	0.731
City (other)	0.621	0.008*	0.436	0.883	0.519	0.002*	0.344	0.783
Village	1	–	–	–	1	–	–	–
**Region (NUTS 1)**								
Central Hungary (HU1)	1.022	0.896	0.737	1.417	0.841	0.500	0.509	1.390
Transdanubia (HU2)	0.788	0.155	0.567	1.094	0.768	0.179	0.522	1.129
Great Plain and North (HU3)	1	–	–	–	1	–	–	–
**Perceived financial status**								
Have no financial problems whatsoever	1.015	0.972	0.450	2.291	1.319	0.586	0.488	3.565
Needs to schedule the expenses	0.916	0.823	0.425	1.976	1.131	0.797	0.442	2.892
The monthly income is just enough	1.000	1.000	0.455	2.196	0.931	0.884	0.359	2.418
Lives month by month. Lacks adequate income	0.723	0.462	0.305	1.713	0.556	0.272	0.195	1.585
Lives in hardship	1	–	–	–	1	–	–	–
**Willingness to get seasonal influenza vaccination**								
Yes	12.009	< 0.001*	7.856	18.358	12.857	< 0.001*	8.161	20.254
No	1	–	–	–	1	–	–	–

*OR* Odds ratio, *AOR* Adjusted odds ratio, *CI* Confidence interval, *NUTS* Nomenclature of Territorial Units for Statistics, *HU* Hungary, *Significant findings (P < 0.05)

### Comparative statistical analysis of influenza vaccination

Table 4 shows the same analyses for seasonal influenza vaccination. Those between the ages 30-39 (*P* = 0.004; AOR = 0.420; 95%CI = 0.233-0.755), 40-49 (*P* < 0.001; AOR = 0.337; 95%CI = 0.221-0.644) and 50-59 (*P* = 0.004; AOR = 0.424; 95%CI = 0.244-0.770) were significantly less willing to get a seasonal influenza vaccine compared to those who are 60 or older. The other demographic factors or the perceived financial status did not significantly influence the responses. On the other hand, the willingness to get a COVID-19 vaccination significantly increased the willingness to get a seasonal influenza vaccination as well (P < 0.001; AOR = 13.265; 95%CI = 8.380-20.977).

**Table 4:** Logistic regression analyses of the differences in the willingness to get seasonal influenza vaccination

Factors	Univariate analysis				Multivariate analysis			
	OR	***P***-value	95% CI		AOR	***P***-value	95% CI	
**Sex**								
Female	0.964	0.818	0.707	1.315	0.923	0.672	0.636	1.339
Male	1	–	–	–	1	–	–	–
**Age**								
18-29	0.742	0.214	0.464	1.187	1.108	0.721	0.630	1.948
30-39	0.333	< 0.001*	0.202	0.552	0.420	0.004*	0.233	0.755
40-49	0.318	< 0.001*	0.200	0.505	0.337	< 0.001*	0.221	0.644
50-59	0.339	< 0.001*	0.205	0.562	0.434	0.004*	0.244	0.770
60 or older	1	–	–	–	1	–	–	–
**Education**								
Primary	1.353	0.120	0.925	1.979	0.997	0.991	0.596	1.667
Secondary	0.852	0.427	0.575	1.264	0.859	0.534	0.531	1.388
Higher	1	–	–	–	1	–	–	–
**Residence**								
Budapest (national capital)	1.083	0.737	0.680	1.724	1.321	0.472	0.618	2.810
City (county capital)	0.868	0.544	0.549	1.372	1.376	0.270	0.780	2.428
City (other)	0.963	0.852	0.646	1.435	1.455	0.131	0.895	2.365
Village	1	–	–	–	1	–	–	–
**Region (NUTS 1)**								
Central Hungary (HU1)	0.877	0.486	0.605	1.270	1.066	0.838	0.580	1.960
Transdanubia (HU2)	0.775	0.187	0.532	1.131	1.018	0.938	0.649	1.596
Great Plain and North (HU3)	1	–	–	–	1	–	–	–
**Perceived financial status**								
Have no financial problems whatsoever	0.552	0.194	0.226	1.353	0.424	0.125	0.141	1.270
Needs to schedule the expenses	0.610	0.243	0.266	1.398	0.521	0.207	0.189	1.434
The monthly income is just enough	0.965	0.933	0.415	2.241	0.833	0.726	0.299	2.318
Lives month by month. Lacks adequate income	0.942	0.900	0.375	2.371	1.125	0.837	0.367	3.453
Lives in hardship	1	–	–	–	1	–	–	–
**Willingness to get COVID-19 vaccination**								
Yes	12.009	< 0.001*	7.856	18.358	13.265	< 0.001*	8.380	20.997
No	1	–	–	–	1	–	–	–

*OR* Odds ratio, *AOR* Adjusted odds ratio, *CI* Confidence interval, *NUTS* Nomenclature of Territorial Units for Statistics, *HU* Hungary, *COVID-19* Coronavirus Disease 2019, *Significant findings (P < 0.05)

### Keyword analysis of COVID-19 vaccination

The most commonly appearing categorical keywords (inferred by the employees of the Nézőpont Intézet Ltd. based on the free text from the participants) related to COVID-19 vaccination is listed in Table 5, Of the ten most commonly occurring words, six were classified as having a positive sentiment, Of the participants whose answer was sorted into a positive category (keyword) related to COVID-19 vaccination, 66,0-81,0% stated to be willing to get that kind of vaccination, and 25,5-36,8% stated the same for seasonal influenza vaccination, On the other hand, of the participants whose reply was sorted into a negative category (keyword) related to COVID-19 vaccination, 25,1-67,1% were willing to get that type of vaccination, and only 17,4-27,4% stated that they were willing to get a seasonal influenza vaccine, When comparing the two types of vaccinations based on the categorical keywords, with the exception of ‘fear’, the willingness to get a COVID-19 vaccination was always significantly higher (*P* < 0,05) compared to the seasonal influenza vaccination.

**Table 5:** Most frequently appearing categorical keywords associated with COVID-19 vaccination and used for the comparative analysis

First 5 keywords mentioned by the participants		COVID-19 vaccination				Seasonal influenza vaccination				***P***-value
Keyword (sentiment)	n	Yes		No		Yes		No		
		n	%	n	%	n	%	n	%	
Mistrust (negative)	207	52	25.1	155	74.9	36	17.4	171	82.6	0.009*
Beneficial (positive)	93	76	81.7	17	18.3	32	34.4	61	65.6	< 0.001*
Protection (positive)	91	70	76.9	21	23.1	32	35.2	59	64.8	< 0.001*
Safety (positive)	76	58	76.3	18	23.7	28	36.8	48	63.2	< 0.002*
Cost (negative)	73	49	67.1	24	32.9	20	27.4	53	72.6	< 0.001*
Uncertainty (negative)	61	19	31.1	42	68.9	8	13.1	53	86.9	0.007*
Fear (negative)	57	20	35.1	37	64.9	15	26.3	42	73.7	0.227
Good (positive)	55	39	70.9	16	29.1	18	32.7	37	67.3	< 0.001*
Health (positive)	47	31	66.0	16	34.0	12	25.5	35	74.5	< 0.001*
Prevention (positive)	36	27	75.0	9	25.0	11	30.6	25	69.4	< 0.001*

*COVID-19* Coronavirus Disease 2019, *Significant findings (*P* < 0.05)

The table shows the most frequent categorical keywords according to the manual processing of the participants replies by the employees of the Nézőpont Intézet Ltd.

### Word co-occurrence networks

**Fig. 1:**
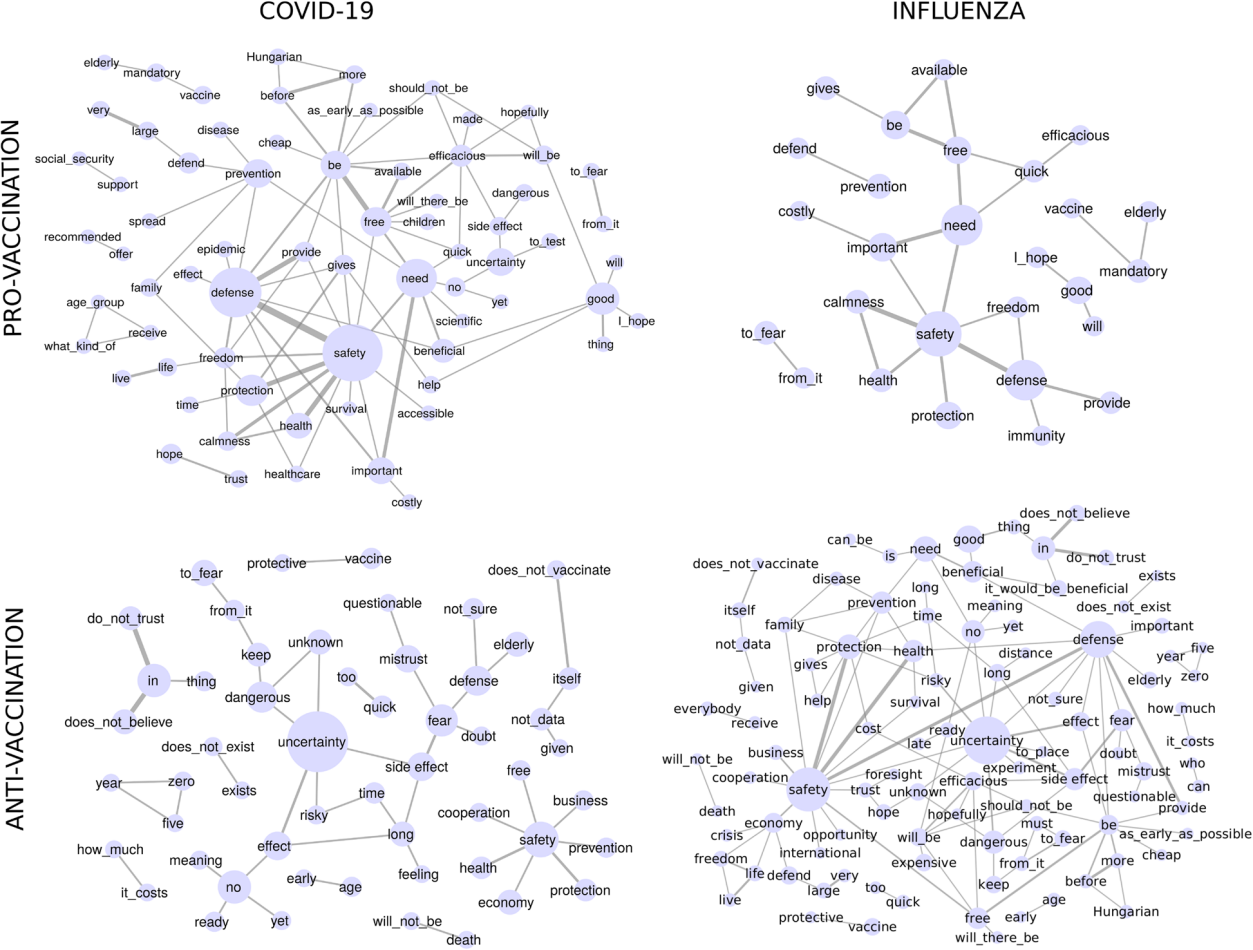
Word co-occurrence networks according to the survey. The width of the connecting lines (links) and the size of the circles (nodes) indicate the frequency, where the required minimal frequency for a link to be taken into account was at least two co-occurrences between the corresponding endpoints

In Fig.1 we depict the word co-occurrence networks based on the free texts obtained from the participants (as shown e.g., in Table 6), collected separately for the four groups described in the Methods section.

**Table 6:** Most frequently mentioned free words associated with COVID-19 vaccination and used for the network analysis

Among top 5 words	COVID-19 vaccination				Seasonal influenza vaccination			
	Yes		No		Yes		No	
	N	%	N	%	N	%	N	%
Safety	48	36.9	17	13.1	23	17.7	42	32.3
Uncertainty	16	14.8	38	35.2	7	6.5	47	43.5
Protection	40	38.5	12	11.5	19	18.3	33	31.7
Necessary	28	36.8	10	13.2	19	25.0	19	25.0
Good	21	36.2	8	13.8	8	13.8	21	36.2
Protection	20	38.5	6	11.5	8	15.4	18	34.6
None	7	15.9	15	34.1	6	13.6	16	36.4
Free	18	40.9	4	9.1	8	18.2	14	31.8
Within	6	14.3	15	35.7	4	9.5	17	40.5
Prevention	17	40.5	4	9.5	6	14.3	15	35.7
To be	17	40.5	4	9.5	9	21.4	12	28.6
Health	14	35.0	6	15.0	5	12.5	15	37.5
Fear	6	15.0	14	35.0	5	12.5	15	37.5
Useful	12	31.6	7	18.4	5	13.2	14	36.8
Dangerous	5	13.9	13	36.1	2	5.6	16	44.4
Useless	0	0.0	17	50.0	0	0.0	17	50.0
Complications	6	17.6	11	32.4	2	5.9	15	44.1
Important	15	46.9	1	3.1	9	28.1	7	21.9
Economy	7	23.3	8	26.7	3	10.0	12	40.0
Effect	4	13.3	11	36.7	4	13.3	11	36.7

*COVID-19* Coronavirus Disease 2019

The table shows the most frequent words used based on the raw input from the participants

For participants who are willing to be vaccinated against COVID-19 (top left panel) the words ‘safety’, ‘defence’, ‘protection’ and ‘need’ are amongst the largest hubs and form a strong backbone around which the rest of the network is organized. Similarly to the above-mentioned backbone, the sentiment of the rest of the nodes is mostly positive (e.g. ‘good’ or ‘help’), where the words with negative connotations (e.g. ‘dangerous’ or ‘side effect’) form only a small subgraph in the network. In contrast, for participants who are not willing to be vaccinated against COVID-19 (bottom left panel), ‘uncertainty’ is the strongest hub, playing the central role in a large network component consisting of mostly negative words. In a separate component with notably strong links, the expressions ‘do not trust’ and ‘does not believe’ indicate prevalent scepticism amongst these participants. The positive words form only a small isolated cluster in this network, centred around ‘safety’. Similar observations can be made when comparing the networks obtained for the participants willing to be vaccinated against influenza (top right panel) and for whom who are not (bottom right panel). The former network consists of mostly positive (or neutral) words, whereas the latter one is mixed, where words with a negative sentiment form the majority, this time displaying a somewhat more entangled and interwoven network structure compared to a COVID-19 related graphs. The notable size difference between the ‘pro-vaccination’ and ‘anti-vaccination’ networks for influenza is caused by the large imbalance between the sizes of the corresponding participant groups, given by 216 people in the ‘pro-vaccination’ group and 624 people in the ‘anti-vaccination’ group.

## Discussion

The current study investigated the willingness of the adult Hungarian population to get a COVID-19 and seasonal influenza vaccine, identified factors that influence vaccine acceptance, and explored the relationship of keywords associated with a COVID-19 vaccine. In this chapter we will first discuss the implications of our findings regarding COVID-19 vaccine hesitancy, then seasonal influenza vaccine hesitancy, then the relationship between the two types of vaccine, and finally, how the keywords were used to describe COVID-19 vaccination.

While only 48.2% of the adult Hungarian population was willing to get a COVID-19 vaccination in our study, this ratio in the United States was between 56.0-68.6% [21–23, 43], 56.6% in Italy [24], 57.7% in Greece [25], 65.4% in Japan [26], 66% in Turkey [27], 72.9% in Finland [28], between 76.0-77.6% in France [29, 30, 33], 79.8% in Canada [34] between 81 and 86% in Australia [31, 32], 83% in the United Kingdom [27], 83.3% in Malaysia [35], and 83.8% in China [36]. However, in Middle Eastern countries the acceptance rate was lower with 21.4% in Lebanon [37], 35.9% in Syria [38], 44.7% in Saudi Arabia [39], and 46% in Egypt [40]. The differences between the countries in regard of COVID-19 vaccine acceptance could be a reflection of how much the citizens trust information from government sources, thus, clear and accurate communication is required by government agencies when dealing with this issue [52].

The unfavourable result regarding the Hungarian population was also prevalent in the report of the European Commission published in December 2020, in which of the 27 member states of the European Union only Bulgaria (34%) and Slovenia (33%) had a higher rejection rate for the COVID-19 vaccination than Hungary (32%) [53]. In this same report, only 49% of the Hungarians were willing to get this vaccination at some point in the future. This means that no meaningful change happened between the survey of which this study was based on and the European Commission’s 2020 report.

By mid-April 4,326,000 people had registered for a COVID-19 vaccine on the official Hungarian registration website, which was 50.6% of all the adult population within the country [54]. This indicates that the data gathered on 2020 August predicted those taking action to get a COVID-19 vaccine very accurately.

Female participants were less willing to get a COVID-19 vaccination, although no significant difference was observed. With the exception of one American and one Malaysian research [22, 35] all other studies found this observation to be significant [21, 25, 27, 30–34, 36–40].

Furthermore, the association between age and the willingness to get a COVID-19 vaccination is unclear. While according to many studies [21, 32, 35, 38, 39] higher age was significantly associated with vaccine hesitancy, other researches came to the opposite conclusion [25, 31–33]. The results of our study are similar to the findings of an American and Canadian studies in which a J-shaped curve described the association between age and the willingness to get a COVID-19 vaccination [22, 34]. It is more or less obvious that the ageing population has been the most endangered risk group during the first wave of the pandemic in Hungary. On the contrary, the younger population survived the SARS-CoV-2 infection with light symptoms or no symptoms at all, and the long-term complications were not known in August 2020. Thus, the data reflect the public opinion on COVID-19 at the time of the study.

The impact of the level of education on the willingness to get a COVID-19 vaccine is also ambiguous. Three American, a French, a Canadian and a Saudi Arabian study found that having a higher education significantly increases the chance that the respondent will be more willing to get a COVID-19 vaccine [21–23, 33, 34, 39]. Two other French and an Egyptian study got the same results, but the difference was not significant [29, 30, 40]. In the Australian, Malaysian and Syrian studies education level had no impact at all [32, 35, 38]. On the other hand, in Turkey and in Greece having a higher education lowered the willingness to get the vaccine [25, 27]. In our study having a higher education significantly increased the willingness to get a COVID-19 vaccine when compared to those having only secondary education, but this difference was not significant when compared to those with primary education.

According to our results living in the capital city of Budapest or in a village was associated with a higher willingness to get vaccinated against COVID-19 compared to those living in any other city in Hungary. Around the summer of 2020 most confirmed COVID-19 infections in Hungary were registered in Budapest, which was widely reported by the media. The fear of getting this infection could have influenced the willingness to get the vaccine in the capital. The reason why those living in villages were more open to getting the vaccine compared to those living in cities remains an enigma.

Better financial status was consistently associated with a significant increase in the willingness to get a COVID-19 vaccine [22, 23, 29, 30]. Although in our study those who stated having the best financial status were also those who were most keen to get a COVID-19 vaccine, still no significant difference was found when comparing the answers to the other categories of perceived financial status.

Overall, due to the differing methodology used in the studies it is challenging to make a firm recommendation on which group should be focused more on when addressing COVID-19 vaccine hesitancy. This, highlight the importance of a unified methodology, in which both the sampling method, confounding factors and statistical analyses should be standardized. Nevertheless, based on our results we recommend to Hungarian policy makers to focus more on the 40-59 aged population with secondary degree living in a city outside of Budapest.

Regarding seasonal influenza vaccination, of the adult Hungarian population only 25.7% were open to get this type of vaccine, which is far lower than the 51.8% of adults living in the United States who actually got vaccinated in the 2019/2020 year [55]. The willingness to get the seasonal influenza vaccine was more favourable among those who are 60 years old or older (38.3%), which is a considerable increase from the 24.1% of Hungarians older than 64 getting the vaccine in 2018 [56]; however, it is not near the recommendation of the World Health Organization, stating that at least 75% of the adults older than 64 should get this vaccine [57].

Despite the fact that the findings are in many cases contradictory, most studies investigating the factors influencing the willingness to get the seasonal influenza vaccination found that being female, older, more educated, and wealthier decrease vaccine hesitancy [41]. In our study education and perceived wealth showed a similar pattern, but no significant differences were observed. Surprisingly, females were more reluctant towards seasonal influenza vaccination, albeit not significantly. Similarly, as with the willingness to get a COVID-19 vaccine, when comparing the oldest group with the middle-aged groups, the willingness to get a seasonal influenza vaccine was significantly higher among the oldest groups; however, this significant value was absent when comparing them to the youngest group, suggesting that there is a J-shaped curve as well.

When comparing COVID-19 and seasonal influenza vaccination acceptance with one another, our findings is similar to the studies conducted in the United States and in Italy [43, 44]. A COVID-19 vaccine is much more accepted compared to a seasonal influenza vaccine. Our analysis highlighted that this observation is true, regardless how we stratify the sample based on the demographic data and perceived income. This shows that the citizens are more open to a vaccine that is related to a serious pandemic and this reaction is uniform through the population. Furthermore, the acceptance of one type of vaccine significantly predicts the acceptance with another, which is in synch with previous studies that shown that having the last seasonal influenza vaccine increases the willingness to get a COVID-19 vaccine [39, 45, 46]. Thus, the overall results suggest that a similar strategy could be utilized to increase the acceptance of getting a COVID-19 vaccine among the general population as for a seasonal influenza vaccine [58, 59].

When analysing the categorical keywords inferred from the free text input from the participants, ‘mistrust’ was the most common category when describing a COVID-19 vaccination. A study conducted in the United States found that ‘mistrust’ (lack of trust) was the second most common reason for rejecting the idea to get a COVID-19 vaccine [22]. A systematic review investigating the relationship between trust and vaccine hesitancy in general had also concluded a strong reversed association between these [60] and studies investigating COVID-19 vaccine hesitancy also emphasize the importance of trust in order to increase acceptancy rates [61–63]. In order to improve trust, governments must utilize clear and accurate communication when addressing any information regarding the disease or vaccination [53].

Similar to the various demographic grouping and the perceived financial status, the willingness to get a COVID-19 vaccine was always more favourable than getting a seasonal influenza vaccination regardless of what kind of words were used, with the exception of ‘fear’, to describe a COVID-19 vaccination. This reinforced the notion that a similar strategy could be utilized to impact vaccine acceptance for both type of vaccines [58, 59].

The word co-occurrence network analysis based on the raw free input text given by the participants showed noteworthy differences between participants who were willing to be vaccinated against COVID-19 and those who were not intending to get a COVID-19 vaccine. The word network for the former group was centred around ‘safety’, ‘defence’, ‘protection’ and ‘health’, and consisted of words with mostly positive sentiment. In contrast, the network for the latter group was organised mainly around ‘uncertainty’, and the majority of the nodes corresponded to words with negative connotations. A previous study conducted in Saudi Arabia demonstrated that holding positive beliefs significantly increase the chance of COVID-19 vaccine acceptance [39]. Therefore, albeit with a different approach, we got the same results.

Finally, this study has some limitations worth mentioning. For example, when narrowing the sample size to those answering each vaccination questions the distribution of the education level changed considerably. However, this reduction was necessary for comparative purposes. Thus, the sample used in the statistical analyses was not representative in this regard. Another noteworthy limitation is that the survey was conducted in August 2020. During that time, there were only hopes for a possible a COVID-19 vaccination, thus, the citizens stated their intention based on a vaccine that not yet existed. There are currently various approved COVID-19 vaccines that the citizens can get [8–11], and a study has already demonstrated that there is preference based on which country a particular vaccine was produced [33]. Further limitation worth mentioning is that many important questions that can influence the willingness to get any form of vaccination were not included [41, 58].

## Conclusion

This study has demonstrated that among the general adult Hungarian population the willingness of getting a COVID-19 vaccine is more favourable compared to the seasonal influenza vaccine. The statement is consistent regardless of demographic grouping, perceived financial status or the usage of words when describing a COVID-19 vaccine. Due to the similarities, the overall results suggest that a similar strategy could be utilized to increase the willingness to get the COVID-19 vaccines among the general population as for seasonal influenza vaccines [58, 59].

## Supplementary Information

**Additional file 1.** The 26 categories created by the Nézőpont Intézet Ltd. The table also contains all the words designated to each category in English. Please note, that due to the translations many words that are different in Hungarian are identical in English. Also, some Hungarian words cannot be expressed with a single word in English.

## Data Availability

The datasets used and/or analysed during the current study are available from the corresponding author on reasonable request.

## Abbreviations

AOR: Adjusted odds ratio
CI: Confidence interval
COVID-19: Coronavirus Disease 2019
Ltd.: Limited company
NUTS: Nomenclature of Territorial Units for Statistics
SARS-CoV-2: Severe Acute Respiratory Syndrome Coronavirus 2
